# Analysis of Epidermal Growth Factor Receptor Related Gene Expression Changes in a Cellular and Animal Model of Parkinson’s Disease

**DOI:** 10.3390/ijms18020430

**Published:** 2017-02-16

**Authors:** In-Su Kim, Sushruta Koppula, Shin-Young Park, Dong-Kug Choi

**Affiliations:** Department of Biotechnology, Konkuk University, Chungju 380-701, Korea; kis5497@kku.ac.kr (I.-S.K.); sushrutak@gmail.com (S.K.); ifresha@nate.com (S.-Y.P.)

**Keywords:** Parkinson’s disease, 1-methyl-4-phenylpyridinium, SH-SY5Y cells, EGFR, microarray analysis

## Abstract

We employed transcriptome analysis of epidermal growth factor receptor related gene expression changes in cellular and animal models of Parkinson’s disease (PD). We used a well-known Parkinsonian toxin 1-methyl-4-phenylpyridine (MPP^+^) to induce neuronal apoptosis in the human neuroblastoma SH-SY5Y cell line. The MPP^+^-treatment of SH-SY5Y cells was capable of inducing neuro-apoptosis, but it remains unclear what kinds of transcriptional genes are affected by MPP^+^ toxicity. Therefore the pathways that were significantly perturbed in MPP^+^ treated human neuroblastoma SH-SY5Y cells were identified based on genome-wide gene expression data at two time points (24 and 48 h). We found that the Epidermal Growth Factor Receptor (EGFR) pathway-related genes showed significantly differential expression at all time points. The EGFR pathway has been linked to diverse cellular events such as proliferation, differentiation, and apoptosis. Further, to evaluate the functional significance of the altered EGFR related gene expression observed in MPP^+^-treated SH-SY5Y cells, the EGFR related *GJB2* (Cx26) gene expression was analyzed in an MPP^+^-intoxicated animal PD model. Our findings identify that the EGFR signaling pathway and its related genes, such as Cx26, might play a significant role in dopaminergic (DAergic) neuronal cell death during the process of neuro-apoptosis and therefore can be focused on as potential targets for therapeutic intervention.

## 1. Introduction

Parkinson’s disease (PD) is the second most long-term degenerative disorder of the central nervous system and is associated with a progressive loss of dopaminergic (DAergic) neurons in the substantia nigra. Although, the pathogenesis of PD is not yet clear, evidence suggests that oxidative stress-induced mitochondrial dysfunction, associated with inhibition of respiratory chain complex I, is one of the main reasons for the onset of PD [1,2]. Experimentally, the symptoms of PD have been achieved using neurotoxin 1-methyl-4-phenylpyridinium (MPP^+^), which selectively and potently inhibits complex I of the mitochondrial electron transport chain [3,4,5,6]. The activation of neuronal cell death pathways involving the mitochondrial dysfunction, as well as increased oxidative stress, most likely represents the process of PD neurodegeneration [7]. Mitochondrial dysfunction results in reduced intracellular adenosine triphosphate (ATP), lower mitochondrial membrane potential, and less endogenous cellular respiration with an augmentation of oxidative stress [8].

Several toxin-induced in vitro models such as 1-methyl-4-phenylpyridinium (MPP^+^), 6-hydroxydopamine (6-OHDA), or rotenone have been widely used in studies to understand the pathogenesis of PD, and, in many aspects, the DAergic neuronal death observed in these conditions is similar to that observed in PD. A reliable cellular model enables large-scale functional screening of genes and interpretation of the molecular signatures through transcriptional profiling. Conn et al. [9] have employed cDNA microarray analysis to characterize the transcriptional response of SH-SY5Y cells to MPP^+^. However, their findings were limited because they examined only 1185 genes for differential expression. Thus, a genome-wide gene expression study is required to identify biological pathways affected by MPP^+^ at the cellular level. Identifying the molecular pathways involved in MPP^+^ toxicity could provide a better understanding of the molecular mechanisms involved in the cell death of PD. Therefore, we compared the expression profiles of MPP^+^ induced genes of an untreated group with a MPP^+^ treatment group, using whole genome expression array (Human Transcriptome Pico Assay 2.0, Affymetrix, Inc., San Diego, CA, USA). Bioinformatics analysis revealed many differentially expressed genes involved in important biological processes. Expression analysis was used to confirm the differential expression of various genes and proteins.

## 2. Results

### 2.1. MPP^+^ Induces Dopaminergic Neuronal Cell Death in SH-SY5Y

**Figure 1:**
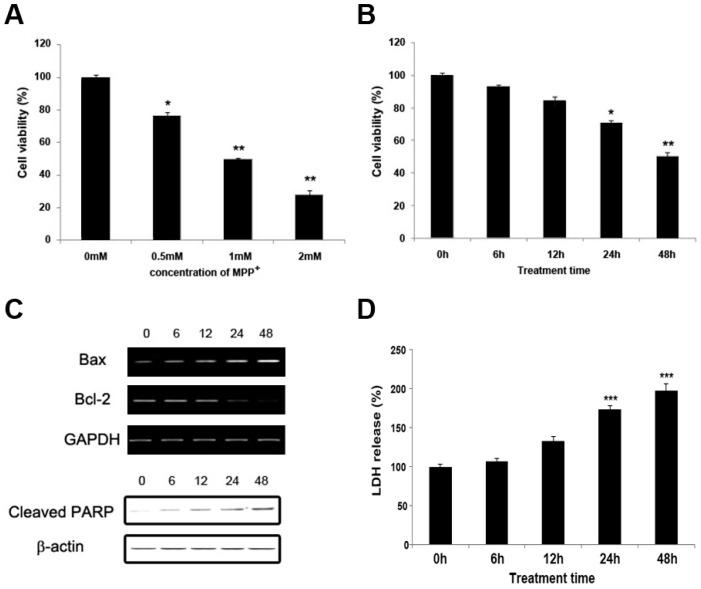

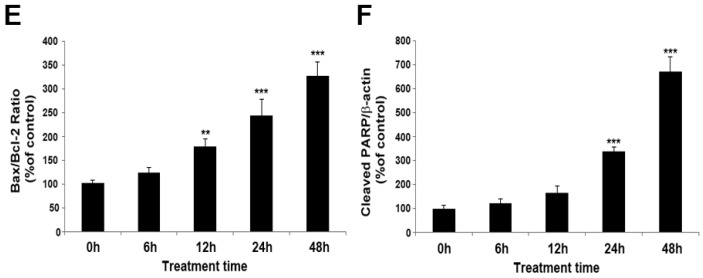
Cell viability (**A**,**B**,**D**) and biochemical markers of apoptosis (**C**,**E**,**F**) in SH-SY5Y cells after treatment for up to 48 h. Cell viability was assessed by the MTT method (**A**,**B**) and LDH assay (**D**) presented as a percentage of untreated controls. The biochemical markers were analyzed by 1mM 1-methyl-4-phenylpyridine (MPP^+^) induced apoptosis after treatment for up to 48 h. (**A**) SH-SY5Y cells were treated with different concentrations (0.5, 1, 2 mM) of MPP^+^ for 48 h; (**B**,**D**) SH-SY5Y cells were incubated for 48 h with 1 mM MPP^+^; (**C**) The biochemical markers of apoptosis were shown by expression analysis; (**E**) The Bax/Bcl-2 ratio was determined; (**F**) The levels of cleaved PARP were quantified by densitometric analysis. Data are expressed as a percentage of values compared to untreated control cultures. Values are mean ± standard error (* *p* < 0.05, ** *p* < 0.01 and *** *p* < 0.005 vs. control group).

Cell viability was measured with MTT (5-diphenyl-tetrazolium bromide) assay based on the conversion of MTT to dark blue formazan crystals by mitochondrial dehydrogenase [10]. Exposure to MPP^+^ showed that concentration and time decreased cell viability when compared to untreated control cells. MPP^+^ at 0.5, 1, and 2 mM significantly (*p* < 0.05 and *p* < 0.01) reduced cell viability when compared to control cells. The higher the concentration of MPP^+^ used, the greater the decrease in cell viability (Figure 1A). The final concentration of MPP^+^ at 1 mM was selected for these trials. As shown in Figure 1B, MPP^+^ was significantly (*p* < 0.05 at 24 h and *p <* 0.01 at 48 h) cytotoxic to SH-SY5Y cells in a time-dependent manner. The percentage of cell viability was approximately 50% after 48 h treatment with 1 mM MPP^+^. Exposure level of cell death was also assayed by LDH (Lactate dehydrogenase) assay, which detected the release of LDH in culture medium. The exposure to 1 mM MPP^+^ for 48 h resulted in a 2.01 ± 0.11-fold increase in the release of LDH when compared to the control cultures (*p* < 0.01 vs. control group) (Figure 1D).

The Bcl-2 family plays a key role in the mitochondrial apoptotic pathway [11]. PARP (Poly ADP ribose polymerase) is an abundant nuclear enzyme with normal function in DNA repair. However, extensive PARP activation can promote cell death [12]. PARP is a biochemical marker for the execution phase of apoptosis. Therefore, we investigated whether MPP^+^ had an apoptotic effect on the expressions of the Bcl-2 family and cleaved PARP in SH-SY5Y cells using RT-PCR and immunoblot analysis. As shown in Figure 1C, the anti-apoptotic molecule Bcl-2 mRNA and the cleaved PARP protein in MPP^+^-treated SH-SY5Y cells were markedly reduced in a time-dependent manner, whereas pro-apoptotic molecule Bax mRNA was increased in a time-dependent manner. Further, there is a time-dependent increase in the Bax/Bcl-2 ratio in cells exposed to 1 mM MPP^+^ (Figure 1E). The ratio of anti-apoptotic versus pro-apoptotic Bcl-2 family proteins regulates apoptosis sensitivity and could be partly responsible in the cell fate decisions [12].

### 2.2. Differential Gene Expression and Pathway from MPP^+^-Treated SH-SY5Y Cells at Two Time Points (24 and 48 h)

To investigate the responses of SH-SY5Y cells exposed to MPP^+^ and understand the underlying molecular mechanisms of MPP^+^ action in SH-SY5Y cells, we used microarrays to determine the gene expression in the two experimental groups; MPP^+^ treatment for 24 and 48 h. Statistically significant (*p* < 0.05) changes in gene expression (fold change ≥2 relative to stimulated cells) were identified by microarray analysis. Many genes were up- or down- regulated by more than two fold by MPP^+^. A total of 6.4% of genes were differentially expressed by MPP^+^ induced cytotoxicity. Among these genes, MPP^+^ treatment t for 24 and 48 h up-regulated 0.5% and 1.3% of the genes in SH-SY5Y cells, respectively, evoking the common expression of 40 genes. MPP^+^ treatment for 24 and 48 h also down-regulated 2.8% and 1.8% of genes, respectively, showing common expression of 0.8% of genes, including the following mitochondrial controlled apoptosis-related genes; BAG1 (BCL-2 binding athanogene-1), BAG5 (B-cell CLL/lymphoma 7C), and BCL-2. Anti-apoptotic molecules were down-regulated by MPP^+^ induced cell death (Table 1).

**Figure 2:**
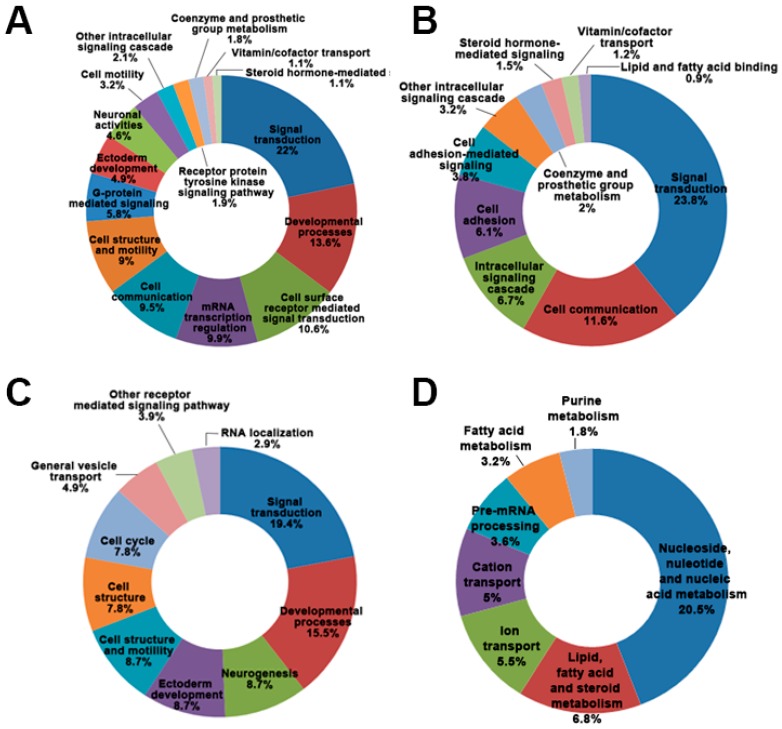
Gene Ontology (GO) biological process classification of differentially expressed genes (DEGs) induced by MPP^+^ treatment. (**A**) Down-regulated genes in SH-SY5Y cells treated by MPP^+^ for 24 h; (**B**) Down-regulated genes in SH-SY5Y cells treated by MPP^+^ for 48 h; (**C**) Up-regulated genes in SH-SY5Y cells treated by MPP^+^ for 24 h; (**D**) Up-regulated genes in SH-SY5Y cells treated by MPP^+^ for 48 h.

Differentially expressed genes (DEGs) were sorted based on the direction of regulation and corresponding Gene Ontology (GO) categories (Figure 2). The use of the DAVID bioinformatics resources (NIAID/NIH, 2008, available online: http://david. abcc.ncifcrf.gov) enabled us to detect gene ontology biological process categories for these differentially expressed genes. The results revealed proteins encoded by these genes associated with several categories (Figure 2). The biggest GO biological process classification of down-regulated genes after MPP^+^ treatment for 24 and 48 h was for genes encoding proteins associated with signal transduction. Between down-regulated genes after MPP^+^ treatment for 24 and 48 h, the common GO biological processes were signal transduction, cell communication, other intracellular signaling cascades, co-enzyme and prosthetic group metabolism, vitamin/co-factor transport, and steroid hormone-mediated signaling.

GO biological process classification of up-regulated genes was distinct between the two MPP^+^ treatment times. The biggest GO biological process classification of up-regulated genes after MPP^+^ treatment for 24 h was for genes encoding proteins associated with signal transduction. However, the GO biological process classification of up-regulated genes after MPP^+^ treatment for 48 h was for genes encoding proteins associated with nucleoside, nucleotide, and nucleic acid metabolism (Figure 2C). These results suggest that signal transduction plays a leading role in MPP^+^ induced DAergic neuronal cell death.

### 2.3. Validation of Microarray Results

**Figure 3:**
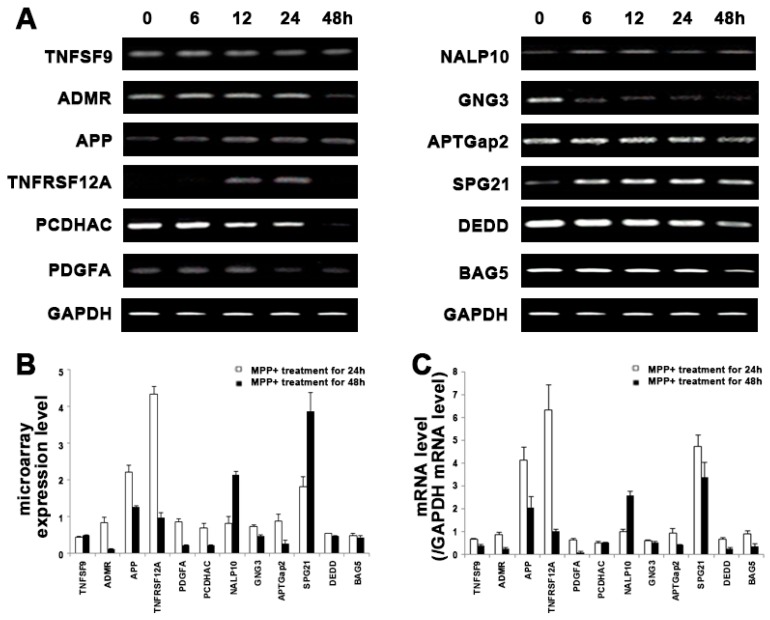
Validation of microarray results. Cells were treated with 1 mM of MPP^+^ for 6 to 48 h. (**A**) Total RNA was extracted and converted to cDNA followed by RT-PCR; (**B**) Bar graphs show microarray results for selected DEGs; (**C**) The levels of mRNA expression were quantified by densitometric analysis. Values are mean ± standard error.

To increase the fidelity of the microarray data, all gene expression values were measured using independent samples at each time point. We selected twelve genes from Table 1 and subjected them to internal validation of expression. The expression patterns of these genes agreed very well with the microarray results (Figure 3). Some of the identified genes were of interest from the context of apoptosis. The TNFRSF12A (fibroblast growth factor-inducible 14 receptor) binds to the soluble TNF-like weak inducer of apoptosis (TWEAK) and induces multiple biological responses, such as proliferation, migration, differentiation, angiogenesis, and apoptosis [13]. In particular, Amyloid β A4 protein precursor (APP) has been recently identified as an integral membrane protein implicated as a regulator of synapse formation, neural plasticity, and iron export [14]. Abnormal metabolism of APP results in β-amyloid accumulation, causing neuronal degeneration in the brains of AD (Alzheimer's disease) patients [15]. Lie et al. [16] reported that over expression of APP will disrupt mitochondrial membrane potentials and redox states.

### 2.4. Analysis of EGFR Pathway-Related Gens in MPP^+^-Induced DAergic Neuronal Cell Death

**Figure 4:**
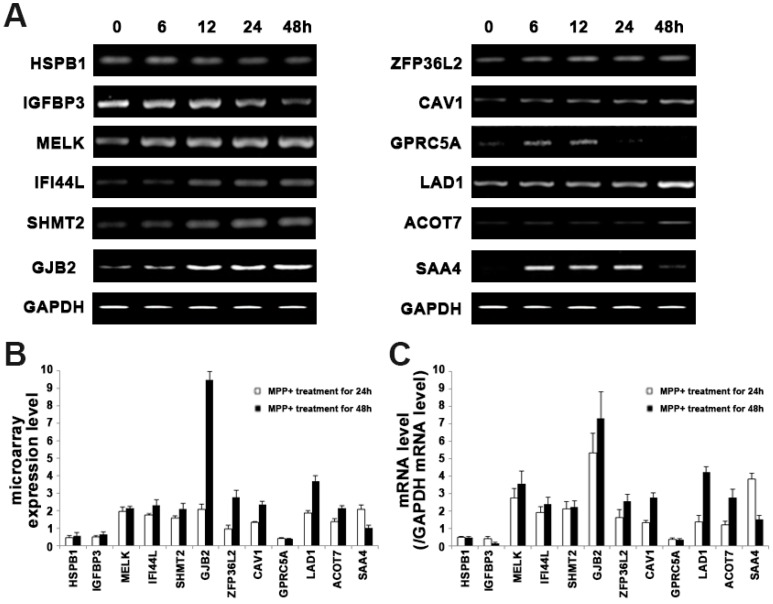
Expression analysis of EGFR related genes in MPP^+^ induced apoptosis. Cells were treated with 1 mM MPP^+^ for 6 to 48 h; (**A**) Total RNA was extracted and converted to cDNA followed by RT-PCR; (**B**) Bar graphs show microarray results for selected DEG; (**C**) The levels of mRNA expression were quantified by densitometric analysis. Values are mean ± standard error.

The use of the KEGG (Kyoto Encyclopedia of Genes and Genomes) pathway database (available online: http://www.genome.jp/kegg/pathway) and NetPath (available online: http://www.netpath.org) enabled us to detect pathways for the differentially expressed genes identified in this study. Various pathways including the EGFR pathway, the Notch pathway, a two-component system, and neuroactive ligand-receptor interaction were found for those differentially expressed genes. In particular, the EGFR signaling pathway-related genes showed significant expression at all time points. We selected twelve genes associated with MPP^+^ induced dopaminergic neuronal cell death (Table 2). Among these genes, three genes (*HSPB1*, *IGFBP3*, and *GPRC5A*) were down regulated (Figure 4). *HSPB1*, *IGFBP3*, and *ZFP36L2* were associated with the p38 MAPK pathway in the EGFR pathway involved in various stresses including reactive oxygen species (ROS), ultra-violet (UV), and toxin [17,18,19]. Other genes (*MELK*, *IFI44L*, *SHMT2*, *SAA4*, *ZFP36L2*, *CAV1*, *GJB2*, *LAD1*, and *ACOT7*) associated with the EGFR signaling pathway were up-regulated in MPP^+^ induced apoptosis (Figure 4). Also two genes (*GPRC5A* and *CJB2*) were associated with the G-protein coupled receptor (GPCR) protein signaling pathway. The EGF signaling pathway regulated the GPCR-related genes [20].

### 2.5. Expression of GJB2 (Cx26) in In Vitro and In Vivo Pakinson’s Model

**Figure 5:**
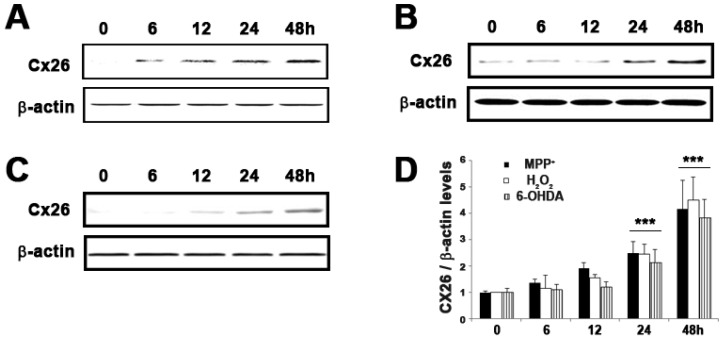
Expression of Cx26 on toxin treated SH-SY5Y cells. SH-SY5Y cells were treated with (**A**) 1 mM MPP^+^, (**B**) 100 µM H_2_O_2,_ and (**C**) 25 µM 6-OHDA for 6 to 48 h. Cell lysate samples (20 µg protein/lane) were measured by immunoblot analysis using Cx26 antibody. (**D**) The levels of CX26 protein were quantified by densitometric analysis. Values are mean ± standard error (*** *p* < 0.01 vs. control group).

To investigate the expression of Cx26 in toxin induced apoptosis and the MPTP (1-methyl-4-phenyl-1,2,3,6-tetrahydropyridine) animal model, we analyzed expression of Cx26 for selected treated times. As shown in Figure 5, there is a significant (*p* < 0.05) and time-dependent increase in the protein expression of Cx26 with the increase in neuronal cell death. After treatment with other toxins such as hydrogen peroxide (H_2_O_2_) and 6-OHDA (100 µM H_2_O_2_ and 25 µM 6-OHDA) for up to 48 h, almost half of the neuronal cell loss was observed, which was similar to MPP^+^-induced cell death. This can result in the generation of reactive oxygen species (ROS). It was well documented that ROS is involved in the pathophysiology of several neurodegenerative diseases, including PD [21,22]. There is a time-dependent increase in the expression of Cx26 protein on toxin treated SH-SY5Y cells, which was similar to that in MPP^+^ treated cells.

**Figure 6:**
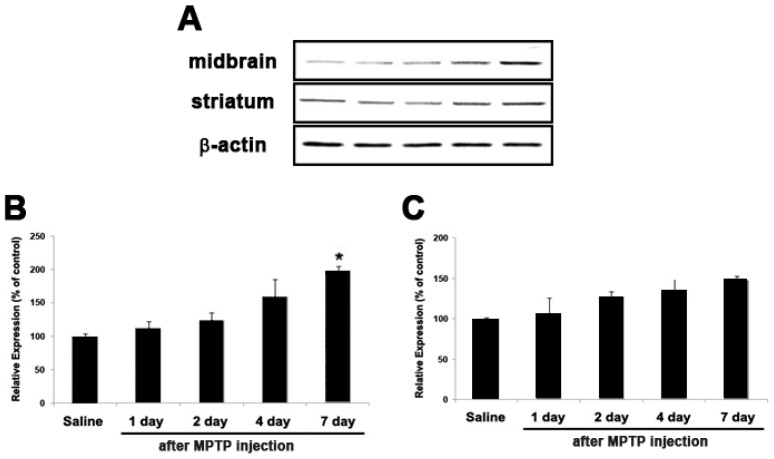
Immunoblot analysis of Cx26 in the midbrain and striatum of a C57BL/6 mouse after sub-chronic MPTP injection. Expression pattern of Cx26 was performed after sub-chronic (**A**) injection of 1-methyl-4-phenyl-1,2,3,6-tetrahydropyridine (MPTP) in C57BL/6 mice. Bar graphs show quantitative data for Cx26 signals that are normalized to the β-actin signal (*n* = 3–4 per group); (**B**) midbrain (**C**) striatum. Values are mean ± standard error (* *p* < 0.05 vs. control group).

The injection of the MPTP neurotoxin has become a widely-used method to investigate the neurodegenerative process. To investigate whether Cx26 levels may be altered in Parkinsonism, we examined the Cx26 protein level in our MPTP-induced mouse PD model. In this model, chronic exposure to MPTP remarkably reduced tyrosine hydroxylase (TH) expression in the substantia nigra pars compacta (SNpc), the same area where a loss of DA neurons occurs in human PD [23]. Figure 6 shows that Cx26 was found in the midbrain and striatum and that the Cx26 protein level was up-regulated in a time-dependent manner. Results indicated that expression of Cx26 was induced by toxins; its role, especially linked on neuronal cell death and neurodegeneration, was evident in both in vitro and in vivo models.

## 3. Discussion

It is well documented that that DAergic neuronal cell death underlies the symptoms of Parkinson’s diseases [24]. The development of a stable and reliable DAergic neuronal injury model is particularly necessary for studying the pathogenesis of PD. Human neuroblastoma SH-SY5Ycells mimic many aspects of the DAergic neuronal cell death observed in PD when treated with neurotoxins such as MPP^+^, 6-hydroxydopamine, and rotenone. We investigated the global gene expression pattern in MPP^+^ treated SH-SY5Y cells. Microarray analysis revealed that 6.4% genes of cellular transcripts were differentially regulated in MPP^+^ induced DAergic neuronal cell death. These differentially expressed genes were sorted based on the direction of regulation. Their corresponding GO categories were identified. The GO terms of down-regulated genes in MPP^+^ induced apoptosis were mostly signal transduction. For up-regulated genes, the GO terms are different. After MPP^+^ treatment for 24 h, most of those up-regulated genes were involved in signal transduction. After 48 h of treatment with MPP^+^, most of the up-regulated genes were involved in nucleoside, nucleotide, and nucleic acid metabolism. The expression results of sixteen differentially-expressed genes identified by microarray analysis were confirmed by RT-PCR, confirming the reliability of the microarray approach.

Genes in the related EGFR signal pathway of DEGs were found to be differentially expressed with different exposure time to MPP^+^. Multiple alterations of gene expressions and signal transduction pathways lie downstream of activated EGFRs, and some of these genes are responsible for cellular defense or apoptosis through expressing either anti- or pro-apoptotic proteins [24,25,26,27]. Some of these genes are responsible for cellular defense or apoptosis through expressing either anti- or pro-apoptotic proteins. Closer inspection of the genes associated with this pathway after MPP^+^ treatment showed that two genes encoding receptor tyrosine kinase (RTK), such as MELK and SHMT2, were differentially expressed (Figure 4). These genes are located very upstream in the EGFR pathway. They affect the entry points controlling this pathway. Therefore, their alteration can be widely propagated throughout the pathway. Also, a previous report indicated increased HSPB1 in PD under conditions of ischemic or thermal stress [28]. HSPB1, which acts through caspase inactivation signaling mechanisms, is known to be neuroprotective and anti-apoptotic [29]. Thus, the increased in expression observed in MPP^+^ induced apoptosis may represent another compensatory response.

Among DEGs of the EGFR related pathway, the connexin proteins are encoded by a multi-gene family, and so far 23 different human Cx genes have been identified [30,31]. The modulation of connexins affected the cell viability or growth, implying that connexins may have an important role in maintaining homeostasis in various organs [32,33]. The connexin is very crucial for the homeostatic regulation in the brain cells and helps with the communication of various electrical and chemical signals for maintaining the neuronal activities [34,35]. Alteration in Cx protein expression may lead to various congenital diseases including hearing loss, which was linked to the Cx protein family [36]. The connexins were regulated by the EGF signaling pathway and apoptosis [37]. Among Cx genes, we have found a Cx26 gene that markedly increased during the MPP^+^ treatment time (Table 2). The alteration in the expression level of Cx26 during MPP^+^-induced apoptosis and whether a similar response was shown in H_2_O_2_- and 6-OHDA-treated SH-SY5Y cells was investigated (Figure 5). Further, we examined the expression pattern of Cx26 in MPTP-intoxicated mice. The Cx26 protein level was significantly increased after MPTP injection, compared with that of saline controls. It is interesting to note that Cx26 that is expressed to colorectal cancer may correlate with Bcl-xL and Bax [38]. Considering the data obtained, our findings suggest that Cx26 might play significant role in DAergic neuronal cell death during the process of neuro-apoptosis. EGFR signaling pathways might also be responsible for DAergic neuronal cell death and therefore can be focused on as potential targets for therapeutic intervention.

## 4. Materials and Methods

### 4.1. Reagents 

MPP^+^, MPTP, and 5-diphenyl-tetrazolium bromide (MTT) were obtained from Sigma Aldrich (St. Louis, MO, USA). Six-well and 100 mm culture dishes were purchased from Nunc Inc. (North Aurora Road, IL, USA). Dulbecco’s modified Eagle’s medium (DMEM), fetal bovine serum (FBS), and other cell culture reagents was purchased from Gibco-BRL Technologies (Rockville, CA, USA). Antibodies against cleaved PARP and β-actin were obtained from Cell Signaling Co., (Boston, MA, USA). All other chemicals used in this study were of analytical grade and obtained from Sigma Chemical Co. (St. Louis, MO, USA) unless otherwise noted.

### 4.2. Cell Culture and Treatments

SH-SY5Y neuroblastoma cells were obtained as described previously [39]. Briefly, cells were grown and maintained in DMEM containing 10% heat-inactivated fetal bovine serum (FBS) and 100 μg/mL penicillin-streptomycin and maintained in a humidified incubator at 37 °C with 5% CO_2_. Following the induction of differentiation with 10 µM all-trans retinoic acid (Sigma-Aldrich, St. Louis, MO, USA) for 7 days prior to treatment, 1 mM MPP^+^, 100 µM H_2_O_2_, and 25 µM 6-OHDA at final concentration were used to treat the SH-SY5Y cells. The total RNAs and proteins were harvested from these cells treated with MPP^+^ for 0, 6, 12, 24, and 48 h, respectively.

### 4.3. Assessment of Cell Viability

Cell viability was measured by using a previously described quantitative colorimetric MTT assay and LDH assay [39]. Briefly, cells seeded at 2.5 × 10^5^ cells/mL in a 96-well plate were treated with 1 mM MPP^+^ for 48 h. The supernatants were removed from each well and the formazan crystals in viable cells were dissolved in DMSO. Optical density was measured at 550 nm using a microplate reader (Molecular Devices, Sunnyvale, CA, USA). Each cell suspension was centrifuged (4000× *g*, 5 min, 4 °C) (Mega 17R, Hanil Scientific Inc., Kimpo-si, Korea) and then the supernatant was collected. LDH activity in the supernatant was performed by using a cytotoxicity assay kit according to the manufacturer’s instructions (Takara Bio, Shiga, Japan). Absorbance was read at 440 nm and cytotoxicity (%) was calculated as:

{(supernatant value − blank value)/[(supernatant value − blank value) + (upper control value − blank value)]} × 100.

Each experiment was performed in triplicate.

### 4.4. Animals and Treatments

The animals were cared for as described previously [40]. Male C57BL/6 mice (age, 8 weeks; weight, 25–30 g) were obtained from Samtako Bio Korea (Gyeonggi-do, Korea) and were used in the present study. All experiments were performed in accordance with the Principles of Laboratory Animal Care (NIH publication No. 85-23, revised 1985) and the experimental procedures were approved by the Institutional Animal Care and Use Committee of Konkuk University (No. KU16171, 6 May 2016). The animals were housed in a controlled environment (23 ± 1 °C and 50% ± 5% humidity) and allowed food and water ad libitum. The room lights were on between 8:00 a.m. and 20:00 p.m. 25 animals were taken and divided into five groups containing five animals each. MPTP sub-chronic (30 mg/kg/day for five days) was administerd to the animals. MPTP was dissolved in saline and prepared just prior to dosing. In the sub-chronic group, the animals were sacrificed at 1, 2, 4, and 7 days to characterize the expression pattern of Cx26.

### 4.5. Microarray Analysis

The experiment was performed using a Human Transcriptome Pico Assay 2.0 (Agilent Technologies, Santa Clara, CA, USA). Microarray analyses were performed as described previously [41]. Total RNA was isolated from cells using TRIzol (Invitrogen, Carlsbad, CA, USA). Extracted total RNA was inspected with a Bioanalyzer 2100 (Agilent Technologies). Microarray hybridization was done according to the manufacturer’s instructions. Fluorescence-labeled cDNA probes were prepared from 5 μg of total RNA by RT primer (Genisphere, Hatfield, PA, USA)-primed polymerization using Super-Script II reverse transcriptase (Invitrogen) in a total reaction volume of 10.5 mL. After reverse transcription, the sample RNA was degraded by adding 1 mL of stop solution (0.5 M NaOH/50 mM EDTA) and incubating 65 μL for 10 min. After two labeled cDNAs were mixed, the mixture was denaturized at 95 °C for 2 min and incubated in a 45 °C water chamber for 20 min. The cDNA mixture was then placed on the cDNA chip and covered by a hybridization chamber. The slides were hybridized in a 62 °C hybridization oven for 12 h. Hybridized slides were scanned with a GenePix 4200 A scanner (Molecular Devices, Sunnyvale, CA, USA). Scanned images were analyzed using GeneSpring 7.3.1 software (Agilent Technologies). To allow algorithmic elimination of bad spots, no data points were eliminated by visual inspection from the initial GenePix image. For signal normalization, positive control genes were spotted onto each slide. The signals of these spots were used for normalization. To determine the background signal intensity, spotting solution was spotted onto each slide. To filter out unreliable data, spots with a signal-to-noise ratio (signal background-background, standard deviation) below 100 were not included in the data. Data were normalized by global, lowess, print-tip, and scaled normalization for data reliability. Genes of interest were those that exhibited a 2-fold change between test and control samples. Data were clustered in groups of genes that behaved similarly across the experiment using GeneSpring 7.3.1 (Agilent Technologies). An algorithm based on Euclidean distance was utilized to separate the genes with similar patterns. The distance cutoff was considered as statistically significant when there was a 2-fold change between time course experiments. The correlation cutoff was 0.95.

### 4.6. Expression Analysis

SH-SY5Y cells (1 × 10^6^ cells/mL) were cultured in six well plates. Total RNA was extracted with TRIzol (Invitrogen). For the reverse transcription-polymerase chain reaction (RT-PCR), 2.5 µg of total RNA from the SH-SY5Y cells was used using First Strand cDNA synthesis kit (Invitrogen). RT-PCR was performed using the cDNA as a template. Nucleotide sequences of the primers used for RT-PCR are shown in Table 3. Glyceraldehyde-3-phosphate dehydrogenase (GAPDH) was used as an internal control to evaluate the relative expressions of specific genes.

The protein expression analyses were performed as described previously [39]. Aliquot (0.1 mL) of RIPA buffer (1× PBS, 1% NP-40, 0.5% sodium deoxycholate, 0.1% SDS containing freshly added protease inhibitor cocktail (Calbiochem, San Diego, CA, USA) was added to the cells cultured in 100 mm plates to obtain the total cell lysate. Cells were scraped, incubated for 10 min on ice, and centrifuged at 14,000 × rpm for 10 min at 4 °C. For the animal experiment, tissues were washed two times with PBS, placed at 4 °C, and homogenized using a 1 mL syringe in lysis buffer (1x RIPA lysis buffer, protease inhibitor cocktail, and phosphatase inhibitor cocktail) and then finally passed through a 311/2 gauge syringe needle and centrifuged at 14,000 rpm at 4 °C for 15 min. The supernatants were collected for further analysis. Protein concentration was determined by the Bio-Rad DC Protein Assay (Hercules, CA, USA). 15 µg of whole cell lysates (cell experiments), or 40 µg of protein (animal experiments) were separated electrophoretically by 10% sodium dodecyl sulfate-polyacrylamide electrophoresis (SDS-PAGE), and the resolved proteins were transferred to polyvinylidene difluoride membranes (Millipore, Bedford, MA, USA). The membranes were incubated for 1 h with 5% skim milk in PBS buffer to block non-specific binding and then incubated with primary antibodies to anti-cleaved PARP, anti-Cx26 (1:1000; Cell Signaling Technology, Boston, MA, USA), and anti-β-actin (1:2000; Cell Signaling Technology). The blots were visualized using the SuperSignal West Pico Chemiluminescent Substrate Detection System (Thermo Scientific, Rockford, IL, USA), according to the manufacturer’s procedure. The optical densities of the antibody-specific bands were analyzed using a luminescent image analyzer, LAS-3000 (Fuji, Tokyo, Japan).

### 4.7. Statistical Analysis

All measurements were performed in triplicates and repeated three times using different samples. Results are presented as the mean ± S.E.M., (*n* = 3). The level of statistical significance was determined by analysis of variance (ANOVA) followed by Dunnett’s *t*-test for multiple comparisons. *p*-values of less than 0.05 were considered statistically significant.

## 5. Conclusions

SH-SY5Yneuroblastoma cells show great potential as a useful research model for research on various CNS diseases such as neurodegenerative diseases, in which DAergic neuronal injury is prominent in the pathophysiology. The DAergic neuronal injury model is particularly necessary for studying the pathogenesis of Parkinson’s disease (PD). We investigated a global pattern of gene expression analysis in MPP^+^ treated SH-SY5Y cells. DEGs were sorted into lists based on the direction of regulation, and corresponding GO categories were identified. The GO term of MPP^+^ induced apoptosis was common in most Signal transductions. Within signal transduction, we investigated the expression of EGFR pathway-related genes. Moreover, we have shown that expression of Cx26 was increased with DAergic neuronal cell death. Therefore EGFR pathway-related genes and Cx26 associated genes might be considered to play an important role, and their regulation may mitigate the neuronal cell death seen in neurodegenerative disorders including PD.

## Figures and Tables

**Table 1:** List of genes with significant (*p* < 0.05) mRNA level changes in MPP^+^ induced apoptosis.

GenBank Accession No.	Gene Symbol	Description	Fold Change		KEGG Pathways	*p*-Value
			24 h	48 h		
NM_001775	*CD38*	ADP-ribosyl cyclase 1	0.49	0.47	Calcium signaling pathway	6.48 × 10^−4^
NM_000957	*PTGER3*	Prostaglandin E2 receptor, EP3 subtype	0.48	0.44		1.58- × 10^−4^
NM_000833	*GRIN2A*	Glutamate receptor subunit epsilon 1 precursor	0.49	0.09		2.62 × 10^−^^5^
NM_004160	*PYY*	Peptide YY precursor	0.49	0.50	Neuroactive ligand-receptor interaction	2.36 × 10^−4^
BC045651	*P2RY5*	P2Y purinoceptor 5	0.43	0.47		1.54 × 10^−4^
BC036030	*GABRG2*	Gamma-aminobutyric-acid receptor γ-2 subunit precursor	0.46	0.43		5.56 × 10^−^^5^
AY358893	*CCL26*	Small inducible cytokine A26 precursor	0.45	0.49	Cytokine-cytokine receptor interaction	4.66 × 10^−^^5^
NM_003811	*TNFSF9*	Tumor necrosis factor ligand superfamily member 9	0.44	0.49		2.05 × 10^−4^
BM555452	*TNFRSF12A*	Tumor necrosis factor receptor superfamily member Fn14 precursor	4.33	0.98		8.36 × 10^−^^5^
X06374	*PDGFA*	Splice Isoform Short of Platelet-derived growth factor, A chain precursor	0.69	0.22		2.43 × 10^−4^
NM_000565	*IL6R*	Interleukin-6 receptor α chain precursor	0.44	0.42		2.23 × 10^−4^
NM_006917	*RXRG*	Retinoic acid receptor RXR-γ	0.46	0.44	Adipocytokine signaling pathway	1.63 × 10^−4^
NM_004457	*ACSL3*	Long-chain-fatty-acid--CoA ligase 3	0.46	0.06		3.37 × 10^−4^
BC030529	*PSMA4*	Proteasome subunit α type 4	2.25	6.75	Proteasome	8.35 × 10^−4^
NM_002790	*PSMA5*	Proteasome subunit α type 5	0.45	0.47		4.39 × 10^−4^
AF097935	*DSG1*	Desmoglein-1 precursor	0.46	0.44	Cell Communication	2.58 × 10^−^^8^
BC034761	*ADMR*	Adrenomedullin receptor	0.84	0.11	G-protein coupled receptor protein signaling pathway	5.33 × 10^−4^
NM_012202	*GNG3*	Guanine nucleotide-binding protein G(I)/G(S)/G(O) γ-3 subunit.	0.73	0.47		3.68 × 10^−4^
BC055286	*A4GALT*	Lactosylceramide 4-α-galactosyltransferase	0.43	0.45	Globoside metabolism	4.71 × 10^−4^
AY643499	*HEXB*	β-Hexosaminidase β chain precursor	0.43	0.49		8.27 × 10^−4^
CR627398	*MAML2*	Mastermind-like 2 (Drosophila)	5.90	2.96	Notch signaling pathway	6.79 × 10^−4^
BC018937	*APP*	Amyloid β A4 protein precursor	2.21	1.27		2.11 × 10^−4^
AK057791	*METTL6*	Hypothetical protein MGC24132	4.60	2.07	Histidine metabolism	4.55 × 10^−^^6^
NM_020997	*LEFTY1*	Left-right determination factor B precursor	0.25	0.30	TGF-β signaling pathway	8.39 × 10^−4^
L04751	*CYP4A11*	Cytochrome P450 4A11 precursor	0.48	0.45	Fatty acid metabolism	6.27 × 10^−4^
BC073828	*PPM1J*	Protein phosphatase 2a, catalytic subunit, isoform	0.46	0.45	Tight junction	2.61 × 10^−4^
NM_001015049	*BAG5*	B-cell CLL/lymphoma 7C	0.49	0.43	Anti-apoptosis	7.38 × 10^−4^
NM_004323	*BAG1*	BCL-2 binding athanogene-1	0.48	0.32		6.83 × 10^−4^
NM_000633	*BCL2*	B-cell CLL/lymphoma 2	0.48	0.21		2.1 × 10^−^^6^
AK001497	*DEDD*	Death effector domain-containing protein	0.54	0.47	Induction of apoptosis	7.85 × 10^−4^
NM_031882	*PCDHAC1*	Protocadherin α subfamily C, 1	0.85	0.23	Cell adhesion	3.67 × 10^−^^5^
CR622836	*NALP10*	NACHT-, LRR- and PYD-containing protein 10	0.81	2.13	Unknown	8.90 × 10^−^^6^
AK075382	*ATP6AP2*	Vacuolar ATP synthase membrane sector associated protein M8-9	0.88	0.26		3.47 × 10^−4^
NM_016630	*SPG21*	Spastic paraplegia 21	1.81	3.88		1.24 × 10^−^^4^
BC039110	*SMNDC1*	Survival of motor neuron-related splicing factor 30	2	2.7		1.54 × 10^−^^5^

**Table 2:** List of genes identified in EGFR signaling pathway related genes.

GenBank Accession No.	Gene Symbol	Description	Fold Change		*p*-Value
			24 h	48 h	
BM907768	*HSPB1*	Heat-shock protein β-1	0.48 ± 0.09	0.58 ± 0.16	3.47 × 10^−^^4^
NM_001013398	*IGFBP3*	Insulin-like growth factor binding protein 3 precursor	0.49 ± 0.08	0.64 ± 0.14	1.54 × 10^−^^5^
NM_014791	*MELK*	Maternal embryonic leucine zipper kinase	1.96 ± 0.24	2.15 ± 0.09	1.18 × 10^−^^4^
AL832618	*IFI44L*	interferon induced protein 44-like	1.76 ± 0.09	2.31 ± 0.33	1.20 × 10^−^^4^
AK055053	*SHMT2*	Serine hydroxymethyltransferase	1.59 ± 0.12	2.12 ± 0.29	3.47 × 10^−^^4^
NM_004004	*GJB2*	Connexin 26	2.1 ± 0.30	9.5 ± 0.46	4.12 × 10^−^^5^
NM_006887	*ZFP36L2*	Butyrate response factor 2	0.95 ± 0.24	2.77 ± 0.42	4.51 × 10^−^^4^
NM_001753	*CAV1*	Caveolin-1	1.33 ± 0.06	2.38 ± 0.18	1.20 × 10^−^^4^
NM_003979	*GPRC5A*	G-protein coupled receptor family C group 5 member A	0.42 ± 0.04	0.41 ± 0.02	3.66 × 10^−^^4^
NM_005558	*LAD1*	Ladinin 1	1.86 ± 0.14	3.7 ± 0.29	5.54 × 10^−^^4^
NM_181865	*ACOT7*	Cytosolicacyl coenzyme A thioester hydrolase	1.36 ± 0.19	2.14 ± 0.16	1.20 × 10^−^^4^
AY358893	*SAA4*	Serum amyloid A-4 protein precursor	2.09 ± 0.26	1.02 ± 0.16	4.62 × 10^−^^4^

**Table 3:** List of primers used for RT-PCR.

Gene Symbol	Primer	
	Forward	Reverse
*Bcl-2*	ACGACTTCTCCCGCCGCTAC	CCCAGCCTCCGTTATCCTGG
*Bax*	CACCAAGGTGCCGGAACTGA	AATGCCCATGTCCCCCAATC
*TNFSF9*	ACTCGGCCTTCGGTTTCCAG	AAGGGGAGGTCAGCAGCAGG
*ADMR*	GACCGCTATGTCACCCTCAC	GGTGCTGTACGTTTCAAAAGGT
*APP*	AGCTTCTGGCCACTGGGAGG	AGTGGGACATGCGGGGAAGT
*TNFRSF12A*	CTCCTCCAACCACACAAGGGG	TCCCCTCCAAACTCTCCCCA
*PDGFA*	CCAGCGACTCCTGGAGATAGA	CGTCCTGGTCTTGCAGACAG
*PCDHAC1*	TGTGGGGTGGCAGTTTTATGT	GAAAGGAAGCGAAAGTTCCG
*NALP10*	CTACTTACGGGATATGACCCTGT	GCTGGTCCACAAGTTCCAACA
*GNG3*	CCGGTGAACAGCACTATGAGT	GGCATCACAGTAAGTCATCAGG
*ATP6AP2*	AGCTCCGTAATCGCCTGTTTC	GATGCTTATGACGAGACAGCAA
*SPG21*	TCTGACTGGATGGGGTTACCG	GAAGCGCCAAAAAGATGAACTT
*DEDD*	GCACCGCATGTTTGACATCG	TCACGTCCATTTCGGATGAGT
*BAG5*	AATGCAAACCACCCACACCG	ATCAGCCCGAGAGCACACA
*HSPB1*	GTCCCTGGATGTCAACCACT	CTTTACTTGGCGGCAGTCTC
*IGFBP3*	CCAGCTCCAGGTGAGCC	GGTCATGTCCTTGGCAGTCT
*MELK*	CCATGTGCTAGAGACAGCCA	ATGCTACTGGGAGAGAGCCA
*IFI44L*	TCCTAGCCATGTGTCCTTCC	GGCCTACCTCCTCAATTTCC
*SHMT2*	AGACTCAGACTGGGGAAGCA	TCAGTGCCAGCTGGTTGTAG
*GJB2*	CTACTTCCCCATCTCCCACA	GGGAAATGCTAGCGACTGAG
*ZFP36L2*	CACCTTTCATACCATCGGCT	GCAAAGTTGTGGGTCTGGAT
*CAV1*	TGAGATTCAGTGCATCAGCC	AACTTGAAATTGGCACCAGG
*GPRC5A*	TCGTCGCAATGAAGACTTTG	TGGTTCTGCAGCTGAAAATG
*LAD1*	GACCATCTCCTTTCGGATGA	CCCTTGAGACGAAGACTTGC
*ACOT7*	CGTCCTCAGAAAGGAAGTCG	GTTCCTCCACTTGGTCTCCA
*SAA4*	CAGCACAATGAGGCTTTTCA	AGTGACCCTGTGTCCCTGTC
*GAPDH*	GTCAGTGGTGGACCTGACCT	TGTGAGGAGGGGAGATTCAG
